# The emerging role of long non-coding RNAs in tumor-associated macrophages

**DOI:** 10.7150/jca.35770

**Published:** 2019-10-22

**Authors:** Dafei Han, Yilong Fang, Yawei Guo, Wenming Hong, Jiajie Tu, Wei Wei

**Affiliations:** Institute of Clinical Pharmacology, Anhui Medical University, Key Laboratory of Anti-Inflammatory and Immune Medicine, Ministry of Education, Anhui Collaborative Innovation Center of Anti-Inflammatory and Immune Medicine, Hefei, China

**Keywords:** lncRNAs, TAMs, polarization, epigenetics, NF-κB pathway

## Abstract

Tumor-associated macrophages (TAMs) are an important cellular component of the tumor microenvironment (TME) and play an essential role in tumor immunity. Recently, numerous studies have indicated that long non-coding RNAs (lncRNAs) can affect several functions of TAMs. In the present review, we summarize the versatile role of lncRNAs in the polarization, epigenetic modulation, and classic signaling pathways of TAMs, which represent a potential target for tumor diagnosis or treatment.

## Introduction

Long non-coding RNAs (lncRNAs) are a new class of RNAs that are greater than 200 nucleotides in length and have no protein-coding ability 1. Non-coding transcripts have been termed “junk DNA” and “transcriptional noise” in the past few decades, due to their lack of protein coding ability 2. The number of human lncRNAs is about twice that of human protein-coding genes, and compared with the extensively studied small non-coding RNAs (microRNAs or miRNAs) and protein-coding genes, lncRNAs are still in a relatively early stage of the nomenclature, classification and identification. Growing evidence shows that lncRNAs play important roles in both physiological and pathological processes-such as the cell cycle 3, cell differentiation 4, and tumorigenesis 5-7-and other processes, including epigenetics and chromatin remodeling 8, 9. A series of studies have shown that some lncRNAs are involved in tumorigenesis and cancer progression via modulating the tumor microenvironment. LncRNAs have been shown to play a role in colorectal cancer 10, hepatocellular carcinoma 11, pancreatic ductal adenocarcinoma 12, breast cancer 13 and prostate cancer 14 etc. Therefore, elucidating the role of lncRNAs in the tumor microenvironment may help to understand the pathogenesis of tumors and reveal novel therapeutic targets.

The tumor microenvironment (TME) consists of cytokines, growth factors, and chemokines as well as a variety of immune cells induced by tumors. These gradually change during the development of cancer, which in turn promotes proliferation, invasion, angiogenesis, metastasis, immune tolerance, and drug resistance of tumor cells 15, 16. The immune cells in the TME mainly include T and B lymphocytes, macrophages, dendritic cells (DCs), and mast cells. Among these immune cells, the macrophages derived from the embryonic yolk sac, fetal liver, or postnatal myeloid cells are a class of immune cells are characterized by diversity and plasticity, and play an essential role in the immune response 17. According to their polarization state of macrophages, they can be divided into classically activated macrophage (M1) and activated macrophage (M2) phenotypes. Researchers have recently realized that tumor-associated macrophages (TAMs) in the TME are significant to tumors. They interact with adjacent endothelial cells by releasing vascular endothelial growth factor (VEGF), which can promote angiogenesis and blood vessel anastomosis 18. In addition, TAMs can secrete various cytokines and chemokines to recruit regulatory T cells into the TME, directly or indirectly inhibiting the function of CD4^+^ and CD8^+^ effector T cells 19.

Due to the multiple roles that TAMs can play in the TME during the development of various tumors, we attempt here to summarize the function and related mechanism that lncRNAs in tumors by regulating TAMs. We will introduce the versatile role of lncRNAs in TAMs from the following perspectives: polarization, epigenetics, classic signaling pathways, and other regulatory mechanisms.

## The potential cellular mechanisms of interactions between lncRNAs and TAM

### LncRNAs affect polarization of TAM

Macrophages play an important role in innate immunity and TME. According to the polarization state, macrophages can be divided into two types, M1 and M2. M1 macrophages are induced by lipopolysaccharide (LPS) and interferon-γ (IFN-γ), and have inflammatory and anti-tumor properties. Interleukin 4 (IL-4) and Interleukin 13 (IL-13) can induce M2 macrophages, which have anti-inflammatory and tumor-promoting abilities 20. In addition, due to the plasticity of macrophages, the M1 and M2 states are not absolutely immutable. Under certain conditions, the two phenotypes can be mutually transformed. To further clarify how lncRNAs indirectly regulate tumor biology by affecting TAM polarization, several studies regarding the role played by lncRNAs in TAM polarization have been reported in recent years.

Gabrusiewicz *et al.* 21 demonstrated that glioblastoma patients showed a distinguished polarizatsion status of TAM. Two lncRNAs (14orf139 and SNORA25) and one lncRNA (CCDC26) were up-regulated in CD14^+^ blood monocytes of glioma patients. Interestingly, in glioblastoma-infiltrating CD14^+^ TAM, the expression patterns of these three lncRNAs were opposite. In addition, the lncRNAs CCDC26 and RP11-419k12.1 highly expressed in M1 TAM and lncRNA RP11-26E5.1 was M2c TAM. These TAM-related lncRNAs represent potential diagnostic/treatment targets of glioma and require further investigation.

Cyclooxygenase (Cox)-2 protein plays a significant role in regulating the inflammation response in physiology and disease. LncRNA-cox-2, located approximately 50kb downstream of the mouse Cox gene, is inducible in macrophages by Pam3CSK4 (an agonist of Toll-like receptor 1 and 2), LPS, or R848 (an imidazoquinoline compound with potent anti-viral and immune cell-activation properties) in a MyD88- and NF-κB-dependent manner 22. lncRNA-cox-2 had a higher expression in M1 macrophages than non-polarized macrophages and M2 macrophages. In addition, siRNA of lncRNA-cox-2 reduced the expression levels of M1 macrophage markers, including IL-12, inducible nitric oxide synthase (iNOS), and tumor necrosis factor alpha (TNF-α) in M1 macrophages. It also increased the expression of M2 markers, such as IL-10 and arginase-1 (Arg-1), and was found in inflammatory zone 1 (Fizz-1) in M2 macrophages. Furthermore, the proliferation, migration, invasion, angiogenesis, and epithelial-mesenchymal transition (EMT) of HCC cells were repressed when co-cultured with lncRNA-cox-2 knockdown M1 macrophages. Contrastingly, lncRNA-cox-2 knockdown M2 macrophages promoted proliferation and repressed apoptosis of HCC cells. Collectively, these data indicate that lncRNA-cox-2 inhibits tumor growth and immune evasion of HCC cells by inhibiting M2 polarization in macrophages 23.

Coagulation factor X (FX) is a vitamin K-dependent plasma protein that plays an important role in regulating blood coagulation by promoting thrombin production. Studies have shown that FX is overexpressed in glioblastoma multiforme (GBM), and there is a positive correlation between FX expression and TAM density. Interestingly, FX in the TME has the ability to recruit macrophages. In addition, FX promoted M2 polarization of macrophages by increasing phosphorylation and activation of ERK1/2 and AKT in macrophages, eventually boosting GBM development. However, a lncRNA, lncRNA-CASC2c, inhibited the expression and secretion of FX, thereby inhibiting M2 polarization and tumor growth. These findings suggest that CASC2c and FX may serve as potential therapeutic targets of GBM by altering the macrophage polarization in GBM 24.

LncRNA-TUC339, which is primarily derived from HCC exosomes, is involved in the regulation of macrophage activation. Knockdown of lncRNA-TUC339 in THP-1 cells resulted in an increase in the production of pro-inflammatory cytokines and costimulatory molecules and an enhancement of the phagocytotic ability of THP-1 cells. Alternatively, lncRNA-TUC339 overexpression exerted the opposite effect. In addition, lncRNA-TUC339 is highly expressed in M2 macrophages and involved in the M2 polarization of macrophages. LncRNA-TUC339 expression decreased during the transition from the M2 to M1 phenotype. Overexpression of lncRNA-TUC339 in macrophages led to upregulation of M2 markers 25.

Transcribed ultra-conserved RNAs (T-UCRs) - a special class of lncRNAs that regulate mRNA transcription and translation - are a highly conserved RNA sequence between humans and mice 26. One study found that in 30 pairs of HCC training groups and 252 pairs of HCC detection groups, the expression level of T-UCR-uc.306 in HCC cells was lower than that of adjacent tissues. There was a positive correlation between low expression of uc.306 and shorter overall survival. In addition, uc.306 was up-regulated in M1 macrophages. Target predictions indicated that beta-transducin repeat containing (BTRC) protein was a potential target of uc.306. The BTRC could interact with β-catenin (CTNNB1) in the Wnt signaling pathway. In short, macrophage-derived T-UCR uc.306 plays an important role in HCC development by regulating macrophage polarization and the Wnt signaling pathway 27.

Metastasis-associated with lung adenocarcinoma transcript-1 (MALAT1), which is overexpressed in prostate cancer (PC) tissue. PMA/IL-4-induced M2 differentiation of THP1 cells activated MALAT1 expression in PC cell lines. Knockdown of MALAT1 repressed proliferation, invasion, and tumor formation in PC cell lines. M2 macrophages secreted IL-8 could induce MALAT1 via inducing the STAT3 pathway. chromatin immunoprecipitation (ChIP) and luciferase reporter assays showed that STAT3 could bind to the MALAT1 promoter region and transcriptionally stimulate the MALAT1 expression. This report found that the IL-8/STAT3/MALAT1 pathway is function as a key regulators during prostate tumorigenesis 28.

**Figure 1:**
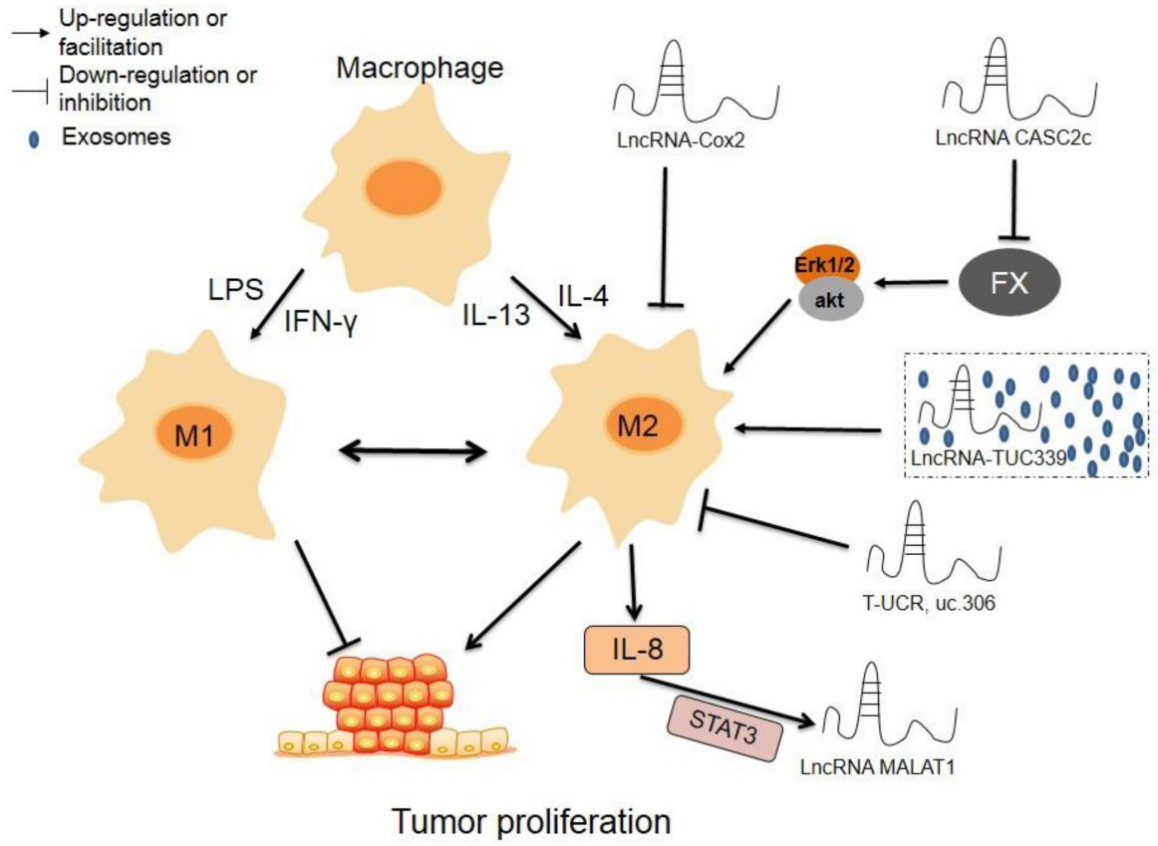
** The regulatory roles of lncRNAs in TAM polarization.** Macrophages can transform into M1 type or M2 type induced by LPS and IFN-γ or IL-4 or IL-13, respectively, and the two phenotypes can transform mutually. LncRNAscox-2 inhibits proliferation and immune evasion of tumor by inhibiting M2 polarization in macrophages. LncRNA CASC2c suppresses the expression and secretion of FX, which lead to decrease phosphorylation and inactivation of ERK1/2 and AKT in macrophages, resulting repressed M2 polarization and decreased proliferation of tumor cells. Exosomes-derived lncRNA TUC339 promotes M2 polarization by increasing the expression of M2 markers. High expression of lncRNA T-UCR, uc.306 is associated with a higher overall survival, which may involve in promoting M1 polarization. M2 macrophages secreted IL-8 could induce the expression of MALAT1 via inducing STAT3 pathway.

Currently, research mainly focuses on two types of immune cells in TME: T cells and macrophages. It is generally accepted that the M1 macrophages have inflammatory and anti-tumor properties, while the M2 macrophages have anti-inflammatory and tumor-promoting abilities. LncRNAs play an important role in the development and progression of tumors, by modulating the M1 or M2 polarization of macrophages, as seen in lncRNA-cox-2, lncRNA-CASC2c, lncRNA-TUC339 and T-UCR, uc.306. These aberrantly expressed lncRNAs can be used as early diagnostic markers for certain cancers or as potential biological targets for cancer therapy (Figure 1).

## The potential molecular mechanisms of interactions between lncRNAs and TAM

### The interaction of lncRNAs and epigenetics affects TAMs

Epigenetics refers to changes in gene expression levels based on non-gene sequence changes such as DNA methylation, histone modifications, chromosomal remodeling, and non-coding RNA (ncRNA) regulation. It mainly controls the function and characteristics of genes by regulating the transcription or translation processes. It also controls and maintains various cellular processes associated with basic health and disease. This section primarily describes how lncRNAs interact with epigenetic regulation in TAMs.

Colon cancer-associated transcript-1 (CCAT1) is abnormally expressed in most human cancer tissues, such as gastric cancer 29 and breast cancer 30. Studies have shown that the levels of CCAT1 and PKCζ, were quite high in M1 macrophages, while miR-148a expression was higher in TAMs. Overexpression of miR-148a promoted the expression of IL-10 and the invasive ability of tumor cells. Downregulation of CCAT1 also promoted M2 polarization of macrophages and invasion of tumor cells by increasing miR-148 expression. In addition, down-regulation of CCAT1 upregulated IL-10 production and tumor cell invasion, an effect that can be eliminated when co-transfected with miR-148a inhibitor. In addition, PKCζ was a target of miR-148a, which could exert its effect by down-regulating the expression of PKCζ. In general, downregulated CCAT1 upregulated the expression of miR-148a and then down-regulated the expression of PKCζ, leading to M2 polarization of macrophages and tumor cell invasion 31.

It has been reported that NIFK-AS1 expression is quite low in TAMs isolated from endometrial cancer patients, but their miR-146a level is increased. Furthermore, overexpression of NIFK-AS1 inhibits M2 polarization (induced by IL-4) in THP-1 macrophages, leading to inhibition of proliferation, invasion, and migration of endometrial cancer cells. In addition, miR-146a interacted with NIFK-AS1. Overexpression of miR-146a attenuated the effect of NIFK-AS1 on macrophage M2 polarization and estradiol production. These findings indicate that lncRNA NIFK-AS1 inhibits M2 polarization of macrophages from the TME of endometrial cancer by targeting miR-146a 32.

LncRNA-NEAT1 (nuclear enriched abundant transcript 1) is a key marker in various tumors, and overexpression of NEAT1 is closely related to the occurrence and development of tumors 33-36. Studies have found that NEAT1 and Arg-1 were highly expressed in thyroid cancer patients, and miR-214 was expressed at a basal level. In addition, NEAT1 could significantly promote the growth and metastasis of thyroid tumor cells *in vitro* and increase tumor size *in vivo*. Knockdown of NEAT1 inhibited migration, invasion, and cell survival of thyroid cancer, accompanied by decreased expression of β-catenin (a direct target of miRNA-214). Finally, NEAT1 could repress the expression of miRNA-214. Together, this evidence reveals that overexpression of NEAT1 accelerates the development of thyroid cancer and promotes the progression of thyroid cancer by regulating the expression of miRNA-214 37.

Chemokines expressed in tumor tissues play an important role in the orientation and differentiation of macrophages by altering the microenvironment of the tumor 38, 39. For example, CCL2 (chemokine C-C motif ligand 2) is produced primarily by a variety of tumor cells, including bladder cancer 40, and is critical for cancer metastasis 41. LncRNA-LNMAT1 (lymph node metastasis-associated transcript 1) epigenetically activated CCL2 expression, resulting in an increased degree of trimethylation of H3K4 (H3K4me3). Upregulated CCL2 recruited macrophages to tumors, thereby accelerating lymphatics. This means that LNMAT1 promoted lymphocyte metastasis of bladder cancer and recruited macrophages via CCL2 42.

**Figure 2:**
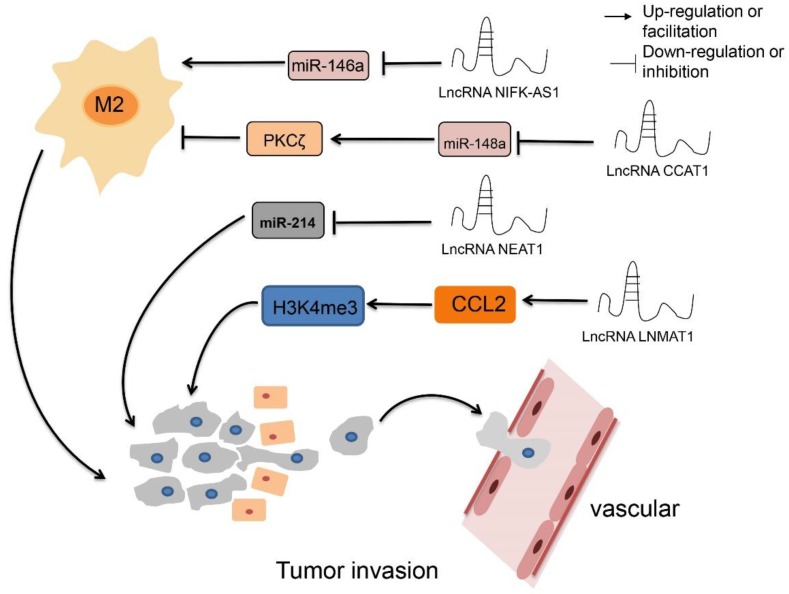
** The regulatory roles of lncRNAs in TAM epigenetics.** LncRNA CCAT1 up-regulates PKCζ by decreasing the expression of miR-148a, resulting inhibiting M2 polarization, while down-regulate LncRNA CCAT1 have the opposite effect. Overexpression LncRNA NIFK-AS1 inhibits M2 polarization by reducing the expression level of miR-146a. LncRNA NEAT1 promotes proliferation and metastasis by inhibiting the expression of miRNA-214. LncRNA LNMAT1 promotes tumor lymphocyte metastasis and recruits macrophages through CCL2.

Apart from the study of genetic mutations, the most productive research area related to tumors is that of epigenetic changes. Epigenetic modifications, without changing the DNA sequence, can control the binding of transcription factors and regulate gene expression. For example, lncRNA-CCAT1 affected the polarization state of TAMs and the invasive ability of tumor cells by regulating the expression of miR-148a. LncRNA-NIFK-AS1 targeted miR-146a, inhibited M2 polarization of macrophage endometrial carcinoma, and affected tumor cell proliferation, invasion, and migration. LncRNA-LNMAT1 epigenetically activated CCL2 expression, promoting recruitment of macrophages and metastases in the TME of bladder cancer. Reduced epigenetic changes during tumorigenesis and development - such as inhibition of DNA methylation induced by lncRNAs and repression of binding of lncRNAs to their transcription factors - lead to restoration of abnormal gene expression, providing a new avenue for research into tumor therapy based on lncRNAs (Figure 2).

### LncRNAs regulate TAMs by affecting classic signaling pathways

#### The NF-κB pathway

Nuclear factor-κB (NF-κB) has been extensively studied due to its important regulatory role in various tumors. It has been recognized that the NF-κB pathway modulates inflammatory factors and anti-apoptotic genes, thereby promoting tumorigenesis.

LncRNA-CamK-A (calcium-dependent kinase activation) was critical in the process of tumorigenesis by participating in the remodeling of the TME via activation of Ca^2+^-activated signaling. CamK-A activated the calcium-dependent NF-κB signaling pathway that led to tumorigenesis, angiogenesis, and macrophage recruitment. In addition, it was quite interesting to notice that inhibition of CamK-A significantly repressed tumor development in a patient-derived xenograft (PDX) model. Clinical studies have also found that CamK-A activated the NF-κB signaling pathway. Furthermore, overexpression of CamK-A is negatively correlated with the survival rate of tumor patients, suggesting that CamK-A may be a potential biomarker and a target for cancer therapy 13.

**Figure 3:**
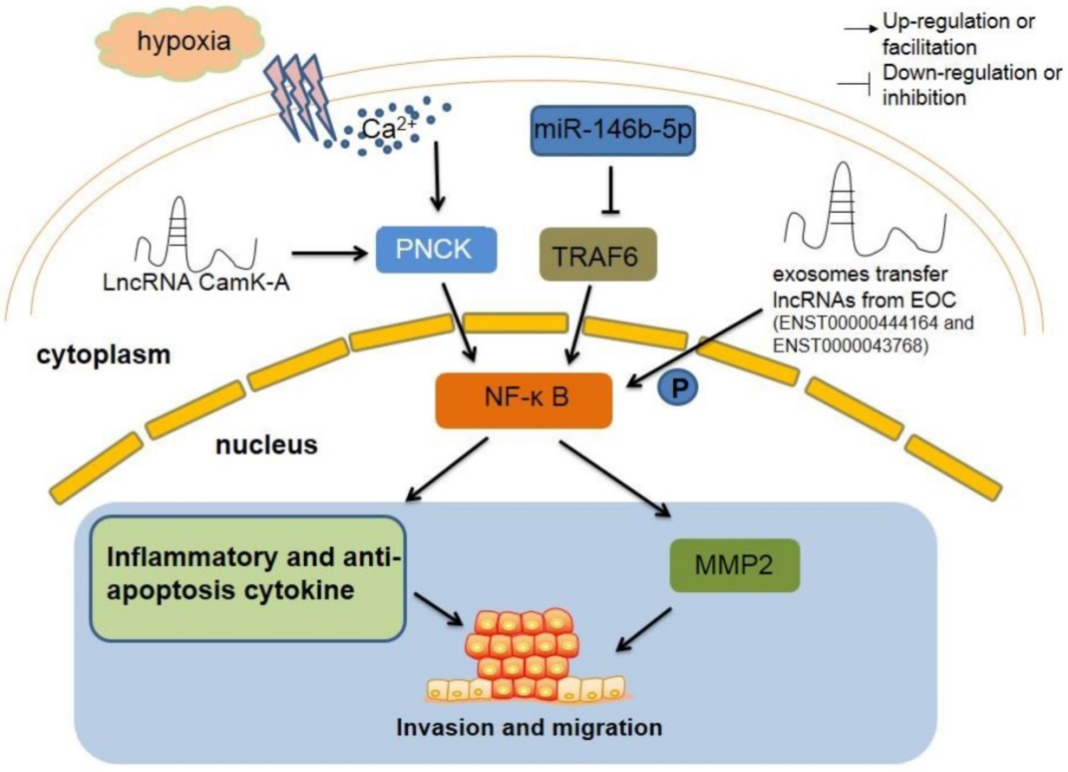
** The regulatory roles of lncRNAs in TAM by regulating classic signaling pathway.** LncRNA CamK-A motivates Ca2+-dependent NF-κB in oxygen deficiency situation, which lead to increase inflammation cytokines and macrophages recruitment, resulting tumorigenesis ultimately. TAM-derived exosomes inhibit the migration of tumor through miR-146b-5p/TRAF6/NF‑κB/MMP2 signaling pathway. However, two lncRNAs from EOC exosome can reverse this suppression. NF-κB, nuclear factor-κB. PNCK, pregnancy upregulated non-ubiquitously expressed CaM kinase. TRAF6, tumor necrosis factor receptor-associated factor 6. MMP2, matrix metalloproteinase2.

The primary TAMs were isolated from epithelial ovarian cancer (EOC) patients and exosomes were collected from primary TAM supernatants. After co-culture with HUVECs, primary TAM-derived exosomes were incorporated into HUVECs and repressed the migration of HUVECs by targeting the miR-146b-5p/TRAF6/NF-κB/MMP2 pathway. In addition, combinational treatment of TAM-derived exosomes and EOC cell line SKOV3-derived exosomes rescued the repression of HUVEC migration. Interestingly, two lncRNAs (ENST00000444164 and ENST0000043768) that from SKOV3-derived exosomes were identified as NF-κB pathway-associated lncRNAs. The potential role of these two EOC exosome-derived lncRNAs is warrant of further investigation (Figure 3) 43.

#### The AKT-P53 pathway

TAMs are also involved in the regulation of other signaling pathways in the TME, such as the AKT signaling pathway. Investigators analyzed altered lncRNAs in breast cancer induced by conditioned medium from cultured human THP-1 macrophages, and they found that a large number of lncRNAs were changed; including lncRNA UCA (urothelial cancer associated 1). Overexpression of UCA1 was detected in human primary breast cancer. Down-regulated UCA1 inhibited AKT phosphorylation and eliminated the invasive ability of tumor cells, which is normally induced by the conditioned medium of macrophages. These results suggest that macrophage infiltration promotes breast cancer invasion by activating lncRNA-UCA1, which may be involved in activation of the AKT signaling pathway 44.

LncRNA-ZFPM-AS1 (ZFPM2 antisense RNA 1) was higher in gastric tumor tissues compared to normal gastric tissues. Overexpression of ZFPM2-AS1 affected tumor size, invasion, grading, and tumor lymph node metastasis. These results were mainly linked to attenuated nuclear translocation of p53. Mechanistic studies have shown that direct binding between ZFPM2-AS1 and macrophage migration inhibitory factor (MIF) reduced the degradation of MIF, which is a potential negative regulator of p53. Knockout of MIF gene eliminated the effects of ZFPM2-AS1 on p53 expression in gastric cancer cells disappeared, indicating that ZFPM2-AS1 is involved in regulation of the ZFPM2-AS1/MIF/p53 signaling pathway. In addition, ZFPM2-AS1 protected MIF from degradation, increased MIF expression, facilitated gastric cancer proliferation, and inhibited apoptosis. These results indicate that ZFPM2-AS1 serves as an emerging oncogene in gastric cancer by protecting ZFPM2-AS1-induced MIF, providing a novel biomarker for diagnosis and a therapeutic target for gastric cancer 45.

Some classic signaling pathways, such as NF-κB, AKT and p53, etc., play important roles in the initiation and development of tumors. Aforementioned lncRNAs, such as lncRNA-CamK-A, lncRNA-UCA1, and lncRNA-ZFPM2-AS1, affect key molecules in these signaling pathways, thereby supporting tumorigenesis. Therefore, restoring these dysfunctional signaling pathways by properly regulating lncRNAs could be a potential direction for tumor therapy.

### LncRNAs affect TAMs through other regulatory mechanisms

LncRNAs play important roles in the TME mainly by affecting macrophage polarization states, signaling pathways, and epigenetic alterations. They also modulate the TME through other mechanisms; for example, MALAT1 was highly expressed in thyroid tumor tissues and promoted angiogenesis of thyroid cancer by regulating expression of fibroblast growth factor 2 (FGF2) expression in TAMs 46. Additionally, in the nutrition insufficient TME, lncRNA-JHDM1D antisense 1 (JHDM1D-AS1) was higher in pancreatic cancer cells and clinical tumor samples than in normal tissues. Overexpression of JHDM1D-AS1 promoted the growth of tumor cells both *in vivo* and *in vitro*, which was mainly related to the increased expression of some tumor-derived angiogenic factors such as human hepatocyte growth factor (hHGF), human fibroblast growth factor 1 (hFGF1), and several inflammatory response genes, including mouse matrix metalloproteinase3 (mMmp3), mouse matrix metalloproteinase9 (mMmp9), mouse S100A8 protein (mS100a8), and mouse S100A9 protein (mS100a9) 47. Therefore, inhibiting tumor growth by inhibiting the activity of JHDM1D-AS1 is a possible strategy for effective treatment of the tumor. In addition, lncRNA Hox antisense intergenic RNA (HOTAIR) plays a key role in the development and metastasis of many cancers, such as hepatocellular carcinoma. Overexpression of HOTAIR upregulated the expression of C-C motif chemokine ligand 2 (CCL2), and increased the proportion of macrophages and bone marrow-derived suppressor cells (MDSCs) in peripheral blood mononuclear cells (PBMCs) 48 indicating that HOTAIR could recruit macrophages and MDSCs to the TME by elevating CCL2 (Table 1) 49.

## Potential consequences and clinical implications of interactions between lncRNAs and TAM

Since TAMs are a significant component of the TME, transformation of the polarization state of TAMs play an important role in the processes of tumor immunology. M1 macrophages can inhibit tumor growth, while M2 macrophages promote tumorigenesis. Therefore, it has been suggested that taking advantage of the M2 to M1 transition may represent a potential therapeutic strategy during tumor treatment. A series of studies have shown that several lncRNAs play a role in this transition, and could be considered as a potential target of TAM polarization. However, the current distinction between M1 and M2 macrophages does not fully embody the complicated characteristics of macrophages, as results indicate that some M1 type macrophage-derived cytokines (such as IL-1β) could also promote tumor growth 50. Therefore, it is essential to more accurately classify M1 and M2 macrophages both *in vitro* and *in vivo*.

## Future direction

In the process of initiation and tumor development, coding genes and non-coding genes may become differentially regulated or undergo mutation in tumor tissues in order to maintain uncontrolled growth. Although some progress has been made in the study of lncRNAs, the understanding of the relationship between lncRNAs and tumors is still at a relatively early stage. Only a few lncRNAs have been well-studied in tumors and their specific structure and function remains to be fully described. Presently, studying the specific structure and function of lncRNAs in tumors is the most pressing issue. Without the proper understanding of the detailed structure and function of lncRNAs, the development of targeted lncRNA-based tumor treatments will be almost impossible. Moreover, the total number of lncRNAs in the genome is extremely large. Therefore, to identify the most essential lncRNAs in various tumors can be a much more complicated task than that of identifying the pathogenic coding gene.

In addition, to fully understand the specific function of lncRNA in TAMs, the improvement of analytical technologies for lncRNAs is also crucial. The use of advanced sequencing and gene editing technology (such as the CRISPR/Cas9 system) to achieve knock-in, knockout, and mutation, can help us to gain a greater understanding of the specific biological function of lncRNAs. However, due to the relatively long length of lncRNAs, it is difficult to investigate the tertiary structure of lncRNAs. Structure-related research into lncRNAs is an emerging field. The actions of lncRNAs largely depend on their tertiary structure. Recently, advances in high-throughput technology have made it possible to detect RNA structures in the genome. Recent developments such as parallel analysis of RNA structures (PARS), fragmentation sequencing (Frag-Seq), selective 2'-hydroxyl acylation analyzed by primer extension, and sequencing (SHAPE-Seq), and dimethyl sulfate-sequencing (DMS-Seq) have successfully begun to determine lncRNA structures across the genome. The combination of these technical methods and computational algorithms can greatly improve the accuracy of lncRNA structure prediction and validation, thereby improving our understanding of the structure and function of lncRNAs, accelerating the elucidation of the role of lncRNAs in tumor progression and other diseases, and enabling lncRNA-based tumor therapies.

## Figures and Tables

**Table 1:** Other lncRNAs in tumor-associated macrophages

LncRNAs	Description of evidence	References
ENST00000444164 and ENST0000043768	two lncRNAs in EOC-derived exosomes (ENST00000444164 and ENST0000043768) reverse the suppression of HUVEC migration by TAMs in the tumor microenvironment.	43
LncRNA UCA1	LncRNA UCA1 phosphorylated AKT and facilitated invasiveness of tumor cells after induced by macrophages conditioned medium.	44
ZFPM2-AS1	ZFPM2-AS1 combined to MIF and reduced the degradation of MIF caused nuclear translocation of p53, which involved in tumor size, invasion, grade, and the stage of tumor node metastasis.	45
LncRNA-MALAT1	LncRNA-MALAT1 facilitated angiogenesis of thyroid cancer by regulating the expression of fibroblast growth factor 2 (FGF2) in TAM.	46
LncRNA JHDM1D-AS1	LncRNA JHDM1D-AS1 increased angiogenic factors derived from tumor, such as, hHGF and hFGF1 and inflammation-responsive genes derived from host, for example, mMmp3, mMmp9, mS100a8, and mS100a9.	47
LncRNA HOTAIR	LncRNA HOTAIR up-regulated the expression of CCL2 and increased the proportion of macrophages and MDSCs in PBMCs, which associated with the development and metastasis of many cancers.	48

## Abbreviations

TAMs: tumor-associated macrophages
TME: the tumor microenvironment
LncRNAs: long non-coding RNAs
DCs: dendritic cells
VEGF: vascular endothelial growth factor
LPS: lipopolysaccharide
IFN-γ: interferon-γ
IL-4: Interleukin 4
IL-13: Interleukin 13
Cox: Cyclooxygenase
Pam3CSK4: an agonist of Toll-like receptor 1/2 (TLR1/2)
R848: an imidazoquinoline compound with potent anti-viral and immune cell-activation properties
iNOS: inducible nitric oxide synthase
TNF-α: tumor necrosis factor alpha
Arg-1: arginase-1
Fizz-1: found in inflammatory zone 1
EMT: epithelial-mesenchymal transition
HCC: hepatocellular carcinoma
FX: coagulation factor X
GBM: glioblastoma multiforme
ERK: extracellular signal-related kinase
T-UCRs: transcribed ultra-conserved RNAs
BTRC: beta-transducin repeat containing
CCAT1: colon cancer-associated transcript-1
NEAT1: nuclear enriched abundant transcript 1
LNMAT1: lymph node metastasis-associated transcript 1
NF-κB: nuclear factor-κB
CamK-A: calcium-dependent kinase activation
PDX: patient-derived xenograft
EOC: epithelial ovarian cancer
UCA: urothelial cancer associated 1
ZFPM-AS1: ZFPM2 antisense RNA 1
MIF: migration inhibitory factor
MALAT1: metastasis-associated lung adenocarcinoma transcript 1
FGF2: fibroblast growth factor 2
JHDM1D-AS1: JHDM1D antisense 1
hHGF: human hepatocyte growth factor
hFGF1: human fibroblast growth factor 1
mMmp3: mouse matrix metalloproteinase3
mMmp9: mouse matrix metalloproteinase9
mS100a8: mouse S100A8 protein
mS100a9: mouse S100A9 protein
HOTAIR: hox antisense intergenic RNA
MDSCs: marrow-derived suppressor cells
PBMCs: peripheral blood mononuclear cells
PARS: parallel analysis of RNA structures
Frag-Seq: fragmentation sequencing
SHAPE-Seq: selective 2'-hydroxyl acylation analyzed by primer extension, and sequencing
DMS-Seq: dimethyl sulfate-sequencing
